# Correlation between Reproductive Hormonal Level and Osteoporosis among Women in Mongolia

**DOI:** 10.5195/cajgh.2015.239

**Published:** 2016-05-06

**Authors:** Unentsatsral Lkhagvasuren, Sarantuya Jav, Battogtokh Zagdsuren

**Affiliations:** 1 Department of Obstetrics and Gynecology, Health Sciences, University of Mongolia, Ulaanbaatar, Mongolia;; 2 Department of Molecular Biology and Genetics, Health Sciences, University of Mongolia, Ulaanbaatar, Mongolia;; 3 Department of Kinesiology, University of Alabama, Alabama, USA

**Keywords:** osteoporosis, post-menopausal women, reproductive hormone, menopause, Mongolia

## Abstract

**Background:**

Postmenopausal osteoporosis is the most common bone metabolic disease associated with low bone mineral density (BMD) and osteopathic fragility fractures, which can lead to significant morbidity. The objective of this study was to investigate the relationship between serum follicle-stimulating hormone (FSH), luteinizing hormone (LH), and estradiol (E2) levels and bone mineral density (BMD) across the stages of menopause in Mongolian women.

**Methods:**

Two hundred sixty participants aged 50.1±4.4 years were enrolled in the study. Blood samples were obtained from each participant and analyzed using ELISA. Data were first stratified and analyzed by bone mineral density status (osteoporotic, osteopenic, and normal) and then by menopause status. Between group differences were analyzed using t-tests, and correlations were assessed using the Spearman rank order test, with Bonferonni correction. The data were analyzed using Statistical Package Statistical Software version 20.0 (SPSS Inc., Chicago, IL). Significance was set at *p*<0.05.

**Results:**

The mean menopausal age was 48.4±3.4, which is comparable to the Mongolian population mean menopausal age. The mean serum estradiol level in the normal BMD group was 18.3±13.1 pg/ml and 15.8±10.7 pg/ml in the osteoporotic group. The mean serum FSH in the normal BMD group was 54.5±44.1 pg/ml and 81.3±34.2 pg/ml in the osteoporotic group. The mean serum LH level in the normal BMD group was 53.1±41.2 and 75.1±26.1 pg/ml in the osteoporotic group. The mean T and Z score were lower in the osteoporotic group. FSH and LH levels significantly differed across menopause stages in that those who were post-menopausal had higher levels compared to those who were pre- or peri-menopausal. Both hormones, FSH and LH, showed weak negative correlations with BMD level, but not E2. There were significant negative correlations between FSH and Speed of Sound (SOS) (r=−0.16; p<0.01), and between osteoporosis with age (r=−0.30, p<0.05) and number of childbirths (r=−0.14 p<0.05).

**Discussion:**

Osteoporosis is a significant problem with associations to hormone levels in post-menopausal women. In our study, mean serum estradiol levels decreased with age, and the mean FSH and LH levels were higher in women of later menopausal stage. Further study is warranted to investigate the bone related studies to establish better statistical references among Mongolian women.

Postmenopausal osteoporosis is the most common bone metabolic disease associated with low bone mineral density (BMD) and osteopathic fragility fractures, which has been associated with significant disability and mortality.^1^ The majority of women spend one third of their lifetime in the postmenopausal period typically accompanied by an estrogen deficit state.^2^ The World Health Organization (WHO) defined osteoporosis as a reduction of 2.5 standard deviations in T-scores below the normal mean for young females at the age of peak bone mass.^3^ A T score between 1 to 2.5 SDs below average indicates osteopenia, which is a pre-osteoporotic state.^3^ The diagnostic measurement of Z-score indicates the bone density relative to patients’ age and sex.^4^ A Z-score below −2.0 is classified as below the expected range for that age, while above −2.0 is considered to be within the expected range for a given age.^4^,^5^ Worldwide, there are nearly 9 million osteoporotic fractures each year, generating a massive burden both to individuals and to health services.^6^ About 13–18% of women over the age of 50 years have osteoporosis and 37–50% have osteopenia in the USA.^7^ Unfortunately, no epidemiologic data have been published about the rate of osteoporosis in Mongolia. Anecdotal evidence suggests that rates in Mongolia are similar to those reported in the USA.

Low estradiol levels are a risk factor for osteoporosis and influences the quality of life for older women.^8^ Estrogen deficiency can lead to excessive bone re-absorption after menopause.^9^ To the best of our knowledge, there are no published studies on the relationship between serum reproductive hormonal level and BMD in postmenopausal women in Mongolia. Therefore, the purpose of this study is to evaluate the correlation between reproductive hormone levels such as follicle- stimulating hormone (FSH), luteinizing hormone (LH), estradiol (E2), and osteoporosis among postmenopausal women in Mongolia.

Osteoporosis research is inadequate in Mongolia due to the lack of reliable diagnostic instruments and poor research infrastructure. Conducting such research is valuable to gerontology, endocrinology, and gynecology fields in Mongolia. Hence, the secondary purpose of this study is to establish a research database for future research in related fields.

## Methods

### Participants

Two hundred sixty women with a mean age of 50.1±4.4 years were included in the study. Participants were recruited from four districts from Ulaanbaatar City of Mongolia. All participants signed informed consents prior to study participation. The study was approved by the ethical committee of Health Sciences University of Mongolia.

Participants were interviewed using a questionnaire developed by the investigators, which included demographics, body mass index (BMI), years since menopause, menarche age, menopause age, and health history. The participants were divided into three different groups based on their BMD value: osteoporosis, osteopenia, and normal. The mean age of the osteoporosis (T score: −2.5), osteopenia (T score: −2.5 to −1), and normal (T score: >−1) group was 54.0±3.9, 51.5±4.3, and 48.5±3.8 years, respectively. The participants were further classified into four menopausal stages: premenopausal, perimenopausal, postmenopausal early and postmenopausal late using the Staging Reproductive Aging in Women (STRAW).^10^ Exclusion criteria included: history of bone disease, metabolic and/or endocrine disorders including hyperthyroidism, hyperparathyroidism, diabetes mellitus, liver disease, renal disease, and medications known to affect bone metabolism (e.g., corticosteroids, anticonvulsants, and heparin sodium). None of the participants had a history of medications for the treatment of osteoporosis, such as active vitamin D^11^, bisphosphonates, selective estrogen receptor modulators (SERM), or calcium.

### Laboratory measurements

Five milliliters of fasting peripheral blood were drawn, centrifuged and kept frozen in the −20° C freezer until assayed. Serum samples were evaluated for levels of E_2_, FSH, LH levels, calcium, phosphorus, and vitamin D by ELISA (Thermo Fisher Scientific, USA) method.

### Bone mineral density measurements

The bone ultrasound Mini-Omni (Sunlight, Beammed, Israel) was used to assess BMD. The parameter of speed of sound (SOS) m/s is used for the analysis.^12^ The SOS refers to the division of transmission time of the sound waves by the length of the body part studied. The forearm and tibia bone scans were performed with the patient on the imaging table using the protocols recommended by the manufacturer. Osteoporosis was defined according to the conventional World Health Organization (WHO) definition.^3^,^13^

### Data analysis

The comparisons of scores between groups were performed using t-tests, and correlations were assessed with the Spearman rank order test, with Bonferonni correction. The data were analyzed using Statistical Package Statistical Software version 20.0 (SPSS Inc., Chicago, IL). Significance was set at p<0.05 and was two-sided.

## Results

### Participant Characteristics

Two hundred sixty (n=260) participants aged 50.1±4.4 years were enrolled in the study. The average BMI was 27.3±5.2 kg/m^2^ and mean age of menopause was 48.4±3.4 years.

### Factors associated with bone density status

The age of menopause (p=0.002), breastfeeding time (p=0.041), and serum vitamin D (p=0.025) were significantly associated with osteoporotic status. However, there were no statistically significant differences of BMI, menarche age, serum calcium, serum phosphorus, and parathyroid hormone levels noted between the three groups. The result of hormone analysis showed serum estradiol levels were lower in the osteoporotic group than in the normal BMD group (normal: 18.3±13.1 pg/ml, osteoporotic: 15.8±10.7 pg/ml). The FSH and LH levels were higher in the osteoporosis group than in the normal BMD group (FSH normal: 54.5±44.1 pg/ml, FSH osteoporosis: 81.3±34.2 pg/ml, LH normal: 53.1±41.2, LH osteoporosis: 75.1±26.1 pg/ml). Clinical and laboratory characteristics of the participants are listed in Table 1.

### Correlation of reproductive hormones with menopausal status

Serum estradiol levels were not significantly different across menopause stages. The FSH and LH levels were higher in the postmenopausal late stage than in the premenopausal stage (Table 2). The study found a weak statistical correlation between FSH and SOS from the ultrasound (r=−0.16, *p*<0.01). There was a significant negative correlation between SOS and BMI (r=−0.306, *p*<0.01). The SOS was significantly different in all four groups (*p*<0.05).

### Analysis of bone mineral density

The average T score was −0.18±0.6 in the normal BMD group, 1.67±0.4 in the osteopenia group, and −3.18±0.6 in the osteoporosis group. The Z score was 0.29±1.2 in normal patients, −1.64±1.3 in the osteopenia group, and −2.17±1.1 in osteoporosis patient group.

**Figure 1.**
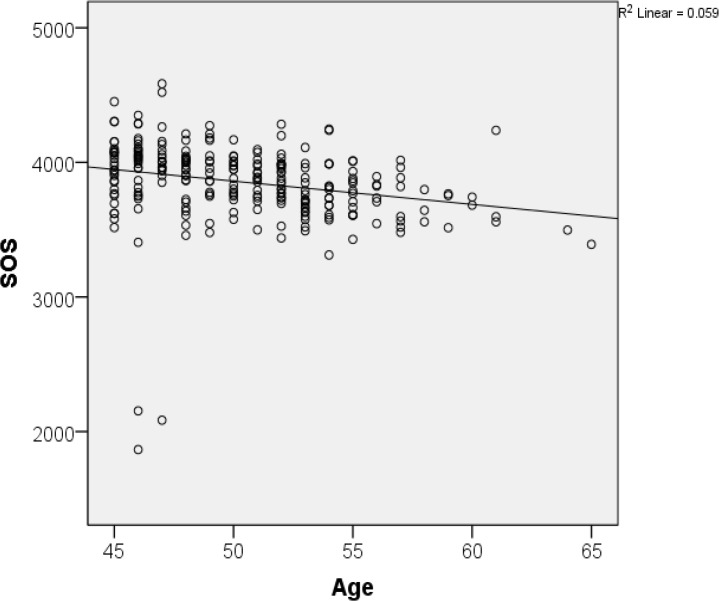
**Correlation between age and bone mineral density**

**Figure 2.**
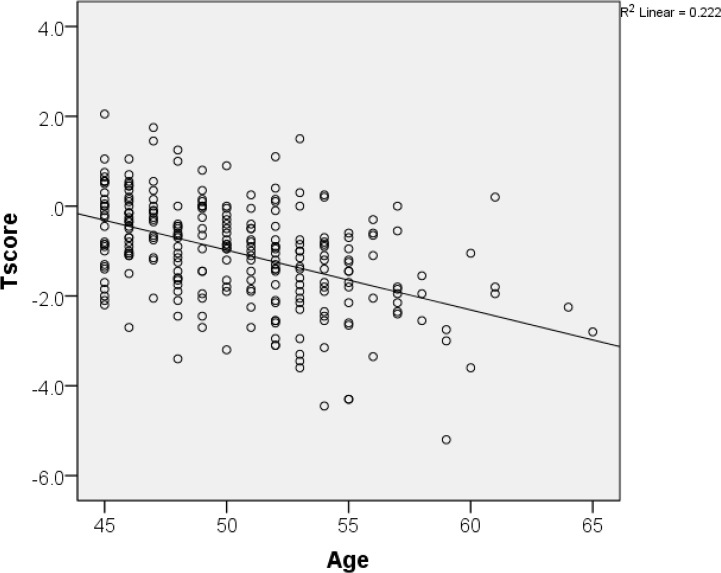
**Correlation between age and speed of sound**

**Figure 3.**
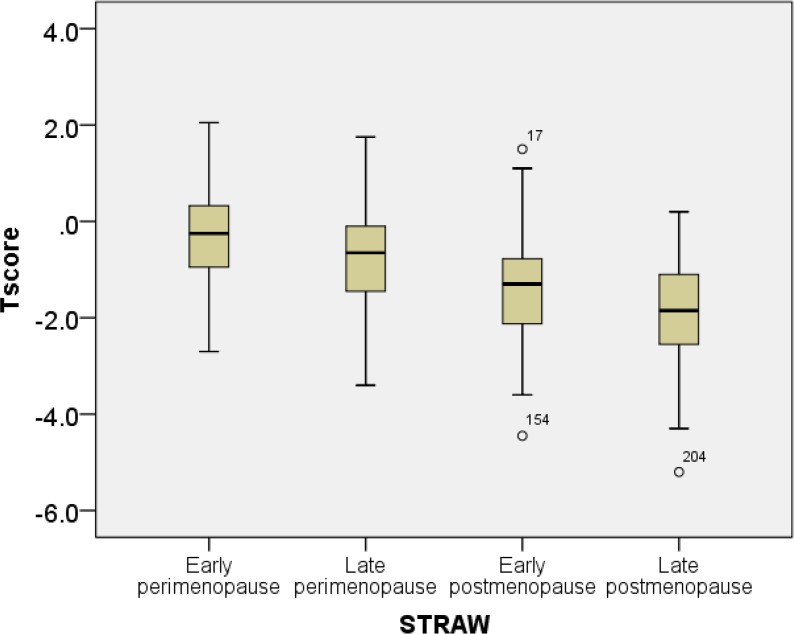
**Box plots of T scores stratified by menopausal status**

**Figure 4.**
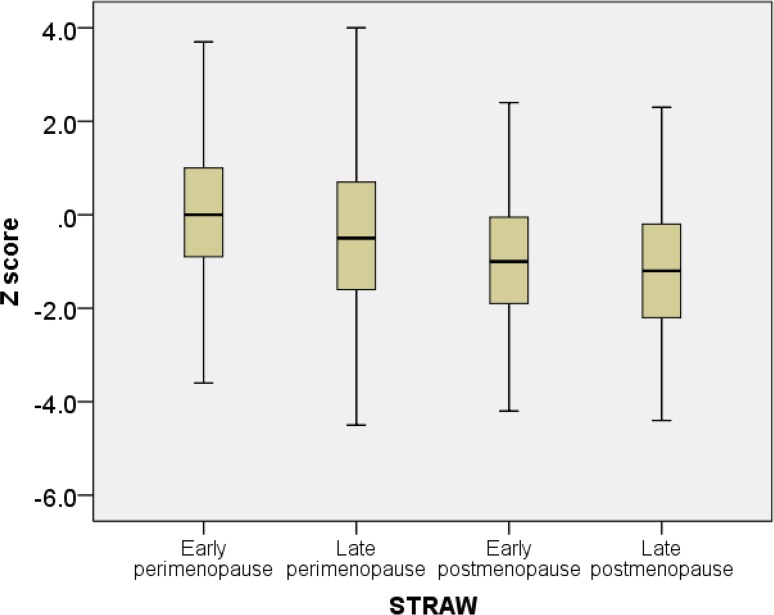
**Box plots of Z scores stratified by menopausal status**

T-score and speed of sound progressively decreased with with age (Figures 1 and 2). The box plots of T and Z scores of the participants stratified by menopausal status are shown in Figures 3 and 4.

## Discussion

The menopausal period is a normal physiological process that is accompanied by various symptoms.^8^ The researchers identified that the average age of onset of the menopause is 48–52, and it is similar in women across various countries.^14^ Recently published study demonstrated that the average age of menopause onset among Mongolian women was 49.3±3.1, 48.6±1.7 and 48.2±2.9 years in 2008, 2010, and 2011, respectively.^15^ Similarly, in our study, the mean age of menopause for our research participants was 48.4±3.4 years.

The current study showed that the rate of osteoporosis was 11.2% and osteopenia was 34.6%, rates that were similar to results of other Asian women from reports of WHO in 2008.^16^ Osteoporosis incidence was higher in women with advanced stages of menopause. In our study, FSH and LH levels significantly differed across menopause stages in that those who were post-menopausal had higher levels compared to those who were pre- or peri-menopausal. Both hormones, FSH and LH, showed weak negative correlations with BMD level, but not E_2_. The result was significant in FSH level and SOS (r=−0.16; *p*<0.01), which was similar to previous studies.^17^,^18^ This could indicate that low BMD in later stages of menopause is associated with serum FSH rather than E_2_. The weak correlation may be due to our small sample size. Other limitations of the current study include: participants were only selected from city areas and rural areas were not sampled, and the BMD was only tested in forearm and tibia. Ideally, the most common fracture sites, such as hips and spine, should be tested in future studies.

This is one of the first studies in Mongolia to determine the correlation between the hormonal levels and menopausal status. We concluded that women from older age groups had lower levels of serum estradiol, which were associated with loss of BMD. However, there was significant correlation between FSH and BMD in more advanced stages of menopause. Further study is needed to investigate the bone metabolism related studies to establish better epidemiological data sources among Mongolian population. Authors would like to encourage similar studies in Mongolia and Central Asia, consistent with the need of Cental Asian Region for better-developed chronic disease research.^19^

## Figures and Tables

**Table 1.** Demographic and laboratory characteristics of participants

Characteristics	Osteoporosis N=30	Osteopenia N=90	Normal N=140	p-value
Mean age	54.0±3.9	51.5±4.3	48.5±3.8	0.433
BMI (kg/m^2^)	28.98±4.0	27.97±5.3	26.55±5.2	0.460
**Age at Menopause (years)**	48.6±3.6	48.2 ±3.5	48.5±3.2	**0.002**
Age at menarche (years)	14.72±1.8	14.47±1.7	14.98±1.9	0.464
Number of birth (n)	3.3±1.5	3.2 ±1.5	2.6±1.2	0.167
Serum calcium (mmol/l)	2.17±0.3	2.18 ±0.2	2.14±0.2	0.077
Serum phosphorus (mmol/l)	3.63±0.9	3.76 ±1.0	3.55±0.9	0.261
Serum PTH (pg/ml)	7.91±4.5	7.62 ±4.2	7.61±5.6	0.565
**Serum VitD (mmol/l)**	13.28±6.1	12.52 ±4.9	12.58±6.1	**0.025**
**Breastfeeding time (yrs)**	2.06±1.2	2.43±1.6	2.38±1.6	**0.041**

* Significant findings are indicated in bold.

**Table 2.** Levels of reproductive hormones (by STRAW) stratified by menopausal status

Stage of menopause	Premenopausal N=63	Perimenopausal N=77	Postmenopausal early N=79	Postmenopausal late N=41
Estradiol (pg/ml)	29.9±3.1	30.7±4.7	34.0±1.7	26.3±7.7
FSH (pg/ml)	26.7±4.7	36.9±4.2	64.6±4.9	61.0±6.3
LH (pg/ml)	20.5±3.6	34.4±3.5	62.3±4.7	59.7±6.9
